# Seroepidemiology of *Toxocara canis* in Children under 14 Years Referring to Laboratories of Sistan and Baluchestan Province in Southeast of Iran

**Published:** 2019

**Authors:** Mahdi KHOSHSIMA-SHAHRAKI, Mansour DABIRZADEH, Hakim AZIZI, Javad KHEDRI, Babak DJAHED, Ali Asghar NESHAT

**Affiliations:** 1. Department of Parasitology & Mycology, School of Medicine, Zabol University of Medical Sciences, Zabol, Iran; 2. Department of Pathobiology, School of Veterinary Medicine, Shahid Bahonar University of Kerman, Kerman, Iran; 3. Department of Environmental Health Engineering, School of Public Health, Iranshahr University of Medical Sciences, Iranshahr, Iran

**Keywords:** *Toxocara canis*, Toxocariasis, Seroepidemiology, Antibodies, Iran

## Abstract

**Background:**

The aim of the present survey was to assess thr seroepidemiologic and parasitological aspects of *Toxocara canis* infection in children under 14 yr old.

**Methods:**

Overall, 963 sera were collected from children in the Sistan and Baluchistan Province, Southeast of Iran during the period from Sep 2015 to Jun 2016. IgG antibody against *T. canis* in the subjects’ sera was evaluated using the commercial ELISA kit.

**Results:**

Anti-*Toxocara* IgG were detected in the serum of 17 (1.7%) of the participants. In the examined children, the highest presence of anti-*Toxocara* antibodies was 2.1% (9/418) in 6-10-yr olds, which was higher than other age groups (*P*<0.05). Anti-*Toxocara* antibodies were significantly higher in males (2.4% or 12/492) than in females (1.1% or 5/471) (*P*<0.03). Highest serological prevalence of *T. canis* occurred in tribes (5.5% or 4/69), followed by rural areas (0.9% or 7/757), while in the urban area it was 0.1% (6/163) (*P*<0.01). A significant association was seen between the serological prevalence of *T. canis* and laboratory findings such as eosinophilia (*P*=0.001) and red blood cell count (*P*=0.02).

**Conclusion:**

Seroprevalence of *Toxocara* infection is high among children living in the poor regions of southeast Iran.

## Introduction

Toxocariasis is an important parasitic disease caused by the ascarid larvae of *Toxocara* genus, which are intestinal nematodes (1, 2). Their definitive hosts are domestic dogs and cats. They develop in the intestines and occur mostly in young animals (3). The most common parasitic agents of toxocariasis are *Toxocara canis* and, less frequently, *Toxocara catis* (4).

Adult female *T. canis* worms can lay as many as 200,000 eggs per day. Non-embryonated *Toxocara* eggs are passed through dog feces, become infectious in suitable environments (temperatures of 10–35 °C and high soil humidity), and can remain infective in the soil for many years (5, 6). Human infection can occur through the ingestion of embryonated eggs from contaminated sources (e.g. soil and earthworms), dirty hands, uncooked vegetables, and paratenic hosts (7). The larvae can then penetrate the intestinal wall and migrate for months through different organs until they are overcome by the human inflammatory reaction and finally die (6).

Human infection can result in a variety of syndromes with different clinical manifestations. Three commonly described syndromes are visceral larva migrans (VLM) with eosinophilia, pulmonary disorders, hepatomegaly, hyperglobulinemia, pneumonitis, and neurological disorders, ocular larva migrans (OLM) that leads to severe chorioretinitis, uveitis, strabismus, and even blindness, and occult or covert toxocariasis (CT) (1,6). About 76% of the world’s population of stray dogs are infected with toxocariasis. This is an important epidemic factor in nature (5). Although there have been a number of studies on the prevalence of *T. canis* in stray dogs and soil contamination due to *T. canis* eggs, there is a gap in our information about the presence of *T. canis* in children and others study that it may be related to regional climate as humidity, dryness, behaviours and laboratory methods for detection, people’s attitudes toward pets. (4,8,9).

Thus, this study, aimed to assess the seroprevalence of toxocariasis infection among children under 14 in rural, urban, and tribal laboratories.

## Materials and Methods

### Field study area

**Fig. 1:**
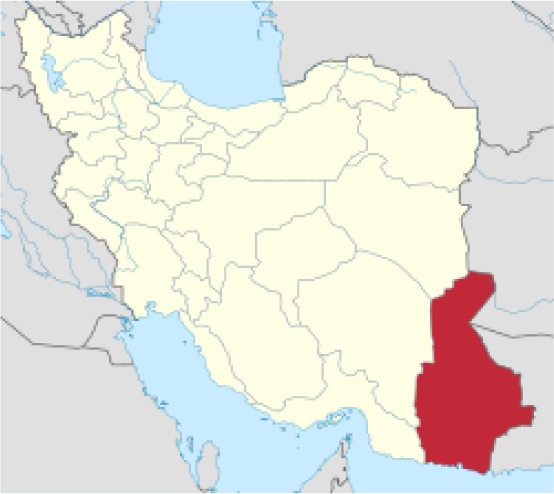
Sistan and Balucheistan Province and its counties (from Wikipedia)

The present study was performed in Sistan and Baluchistan Province in southeast Iran. It is located between latitudes 25°3′ and 28°31’ north and longitudes 58°48’ and 63°19’ east. This province lies on the border with both Afghanistan and Pakistan. It has a hot and dry climate and is moderately dry in the winter. The average annual rain in Sistan and Baluchistan is reported at around 59 mm and the relative humidity is at ~ 40% (10) (Fig.1).

### Sample size

Overall, 963 children (471 female and 492 male) under 14 yr in different age groups (1–5 yr old, 6–10 yr old, and 11–14 yr old) were selected from Sep 2015 to Jun 2016 from private, public, and tribal laboratories of Sistan and Baluchestan. The sample size was calculated as 963 using parameters based on a previously published study (11). Data pertaining to each examined participants were collected and recorded in individual files.

### Determination of serum antibodies level against Toxocara

For titration of IgG antibody against *Toxocara* antigens in the laboratory, a blood sample of 4 ml was collected from each child. The collected sample was divided into two tubes—one for blood cell count and the other for measuring anti-*Toxocara* IgG by ELISA method. The tubes were stored at −20 °C until used. All specimens were tested using a commercial enzyme immunoassay kit (IBL Germany) in accordance with the manufacturer’s instructions.

### Statistical analysis

For the analysis of data, descriptive statistics for qualitative data with 95% confidence interval (95% CI) was used to determine the effect of the mentioned risk indicators on the prevalence of infection. A *P*-value less than 0.05 was considered to be statistically significant. Data were analyzed using Stata, ver. 11.2.

### Ethical Approval

The study protocol was performed according to the Helsinki declaration and approved by Ethics Committee of Iranshahr University of Medical Sciences (Permit Number: IR.IRSHUMS.RES.1394.10). Informed written consent was obtained from all patients before they participated in the study.

## Results

Out of 963 serum samples from patients, 17 (1.7%) were positive for anti-*Toxocara* antibodies. Mean antibody titer was 2.2 (± 1.1) in the studied cases. In the examined children, the presence of anti-*Toxocara* IgG was 2.1% (9/418) in 6-10-yr-old, which was higher than that in the other age groups (*P*<0.05). Anti-*Toxocara* antibodies were significantly higher in males (2.4%, 12/492) than in females (1.1%, 5/471) (*P*<0.03). The influence of different characteristics of participants, such as city of residence (*P*=0.002), gender (*P*=0.03), consumption of vegetables (*P*=0.03), and living place (*P*=0.01) on the prevalence of toxocariasis antibody was significant (Tables 1–3). A significant association was seen between the serological prevalence of *T. canis* and laboratory findings such as eosinophilia (*P*=0.001) and red blood cells count (*P*=0.02) (Table 4).

**Table 1:** Seroepidemiology of *Toxocara* according to city&area of residents of the participants

***Characteristics***		***Negative serology N (%)***	***Positive serology N (%)***	***P-value***
City of residence	Zahedan	493(99.4)	3(0.6)	0.002
	Iranshahr	189(97.9)	4(2.1)	
	Khash	95(97.9)	2(2.1)	
	Nikshahr	94(95.9)	4(4.1)	
	Saravan	92(95.8)	4(4.2)	
Area of residents	Urban	163(99.9)	6(0.1)	0.01
	Rural	757(99.1)	7(0.9)	
	Tribes	69(94.5)	4(5.5)	

**Table 2:** Seroepidemiology of *Toxocara* according to sex & age of the participants

***Characteristics***		***Negative serology N (%)***	***Positive serology N (%)***	***P-value***
Gender	Male	492(97.6)	12(2.4)	0.03
	Female	471(98.9)	5(1.1)	
	Employee	88(98.9)	1(1.1)	
	Self-employed/jobless	765(98.3)	13(1.7)	
Age group (yr)	1–5	353(98.6)	5(1.4)	0.05
	6–10	418(97.9)	9(2.1)	
	11–14	192(98.5)	3(1.5)	

**Table 3:** Seroepidemiology of *Toxocara* according to some socio-economic factors

***Characteristics***		***Negative Serology N (%)***	***Positive Serology N (%)***	***P Value***
Dog Exposure	Yes	242(99.2)	2(0.8)	0.2
	No	721(98)	15(2)	
Vegetable Consumption	Once A Day	399(97.1)	12(2.9)	0.03
	Every Other Day	483(99.2)	4(0.8)	
	One Serving A Week	81(98.8)	1(1.2)	
Education Level Of The Parents	Illiterate	505(99.9)	8(0.1)	0.4
	Diploma	382(98.2)	7(1.8)	
	Academic	76(97.4)	2(2.6)	
Father’s Job	Related To Animals	110(97.3)	3(2.7)	0.6
	Employee	88(98.9)	1(1.1)	
	Self-Employed/Jobless	765(98.3)	13(1.7)	

**Table 4:** Seroprevalence of *Toxocara* according to the laboratory results

***Lab results***		***Negative serology N (%)***	***Positive serology N (%)***	***P-value***
Eosinophil (%)	0≤5	338(100)	0(0)	0.001
	≥6	625(97.4)	17(2.6)	
Hemoglobin	Normal	704(98.7)	9(1.3)	0.5
	Low	259(97)	8(3)	
WBC count	Normal	782(98.2)	14(1.8)	0.8
	high	181(98.4)	3(1.6)	
RBC count	Normal	811(98.7)	11(1.3)	0.02
	low	152(96.2)	6(3.8)	
Platelet	Normal	854(98.5)	13(1.5)	0.3
	low	109(96.5)	4(3.5)	

## Discussion

Toxocariasis is a global health problem in the general population and children are at the highest risk (2). Additionally, there has been an increase in the number of stray dogs and cats in recent years. This can lead to zoonotic diseases that can even cause death in humans (4). Toxocariasis is between 2.7% and 29.3% in different areas of the country of Iran (8, 12). In our study, the overall seroprevalence of *Toxocara* infection in patients was 1.7%. There are publications on the prevalence of *T. canis* among children in different parts of Iran and other countries (in Iran: 2% in Shiraz, 19% in Ahwaz, and 2.7% in Zanjan; among other countries: 48.4% in Brazil, 37.9% in Argentina, and 12.95% in Turkey) (13–16, 3). The difference in the prevalence of toxocariasis could be attributed to cultural habits, food habits, geographical location, climate condition, and temperature (17).

The titer of anti-*T. canis* IgG was 2.2% in our study group and was compatible with previous studies. In a report from the northwestern part of Turkey, including Eskisehir, Bilecik, Kutahya, and Afyon, Dogan studied seroprevalence rate of *Toxocara* antibody in children and detected that 16.97% and 0.71% were a positive titer of anti-*Toxocara* antibodies from rural and urban areas respectively (14). It is very difficult to explain the exact differences in anti-*Toxocara* antibodies in children from different areas. However, a combination of factors, including some forms of cultural habits, method of cooking food, climates and soil condition, vegetative distribution, and humidity level could influence *Toxocara* antibodies (17, 5).

In the current investigation, the *Toxocara* infection rate was found to be higher in tribal areas than rural and urban areas. In contrast, Sadjjadi and Negri reported higher infection rate in urban areas than in rural areas (18,19). Cultural habits, climate condition, and a closer connection to dogs affect the prevalence of *Toxocara* infection in different areas (15, 20). The present investigation found that *Toxocara* infection was significantly higher in males than in females (*P*<0.03).

Gender seemed to be an important factor with regard to a positive serology, as shown in our study (16). In Brazil, higher infection rate of *Toxocara* reported in male than in female children (3). However, significantly more females were infected than males (21). The difference in findings may be indicative of the incidence of infection relative to differences in gender-specific behavior and type of games (5). The results of this study showed that a higher rate of *Toxocara* infection was recorded among 6–10-year olds. Our results correspond with another study that reported the maximum infection in children with mean 9.4-year olds in São Paulo Province, Brazil (22). A seroepidemiological study of *T. canis* infection was conducted in children aged 1.4–14.7 yr and reported that the mean age of onset of infection was 7.3 yr (23). This finding was similar to that of the present study. Toxocariasis is considered to be a common parasitic infection among children under 10 yr of age all over the world that may be due to childlike activities and more contact with the ground (22, 9).

Demographic and socioeconomic factors (education level of the parents, the area of residence, and father’s job) may lead to an increase in *Toxocara* seroprevalence. Dogs are the primary hosts and the main vectors of the *Toxocara* parasite. Apart from them, consumption of raw vegetables grown in contaminated gardens, raw or undercooked meat from paratenic hosts, and grounds contaminated with embryonated eggs may be other sources of the disease (8). The risk of *Toxocara* infection is higher among people exposed to dogs and people with low education level (24), which is in agreement with our findings. In this study, we found a significant association between the prevalence of infection with the level of eosinophils and red blood cells. The results of our study are similar to other findings (15), in which the prevalence of infection was significantly associated with high eosinophil levels, while Sarkari et al (16) did not find a significant association between the prevalence of infection with high eosinophil levels. Although blood eosinophilia and a decrease in red blood cells were observed, they were not a consistent characteristic. In the other study, increase in the level of blood eosinophils is reported as one of the most common signals of parasitic infection and decrease in red blood cells, which might indicate a chronic disease (16).

## Conclusion

The results of this study give an overview of the data on the prevalence of toxocariasis in southeast Iran. Future studies should consider the effect of toxocariasis on public health, the role of stray dogs and cats in the epidemiology of toxocariasis, and educational programmes for the prevention and control of *Toxocara*.
